# Salt-tolerant endophytic bacterium *Enterobacter ludwigii* B30 enhance bermudagrass growth under salt stress by modulating plant physiology and changing rhizosphere and root bacterial community

**DOI:** 10.3389/fpls.2022.959427

**Published:** 2022-08-02

**Authors:** Hongjian Wei, Wenyuan He, Ziji Li, Liangfa Ge, Juming Zhang, Tianzeng Liu

**Affiliations:** ^1^College of Forestry and Landscape Architecture, South China Agricultural University, Guangzhou, China; ^2^Guangdong Engineering Research Center for Grassland Science, South China Agricultural University, Guangzhou, China

**Keywords:** salt stress, endophyte, growth-promoting, bermudagrass, bacterial community

## Abstract

Osmotic and ionic induced salt stress suppresses plant growth. In a previous study, *Enterobacter ludwigii* B30, isolated from *Paspalum vaginatum*, improved seed germination, root length, and seedling length of bermudagrass (*Cynodon dactylon*) under salt stress. In this study, *E. ludwigii* B30 application improved fresh weight and dry weight, carotenoid and chlorophyll levels, catalase and superoxide dismutase activities, indole acetic acid content and K^+^ concentration. Without *E. ludwigii* B30 treatment, bermudagrass under salt stress decreased malondialdehyde and proline content, Y(NO) and Y(NPQ), Na^+^ concentration, 1-aminocyclopropane-1-carboxylate, and abscisic acid content. After *E. ludwigii* B30 inoculation, bacterial community richness and diversity in the rhizosphere increased compared with the rhizosphere adjacent to roots under salt stress. Turf quality and carotenoid content were positively correlated with the incidence of the phyla Chloroflexi and Fibrobacteres in rhizosphere soil, and indole acetic acid (IAA) level was positively correlated with the phyla Actinobacteria and Chloroflexi in the roots. Our results suggest that *E. ludwigii* B30 can improve the ability of bermudagrass to accumulate biomass, adjust osmosis, improve photosynthetic efficiency and selectively absorb ions for reducing salt stress-induced injury, while changing the bacterial community structure of the rhizosphere and bermudagrass roots. They also provide a foundation for understanding how the bermudagrass rhizosphere and root microorganisms respond to endophyte inoculation.

## Introduction

Saline-alkali soils are common within terrestrial ecosystems, and salinity affects approximately half of all irrigated land. Typically, salinization increases due to fertilizer overuse and poor water quality (Zhang et al., 2010; Pessarakli, 2014). Plant salt stress (SS) is related to the accumulation of several soluble salts, including calcium chloride, magnesium sulfate, or sodium sulfate, but sodium chloride is the dominant salt-inducing SS within plants (Yu et al., 2013). Soil salinity initially suppresses plant development via osmotic stress, with ionic toxicity appearing later (Liu et al., 2019). SS will produce reactive oxygen species (ROS), thereby inducing oxidative stress (OS) while suppressing plant growth (Essemine et al., 2020). Scorching of leaves and stems, the inhibition of shoot and root developmental, and leaf senescence and abscission, are the major morphological effects induced by SS (Singh and Jha, 2016). In addition, biochemical alterations can involve physiological metabolism, cell oxidation, imbalanced nutritional conditions, and chlorophyll degradation (He et al., 2019; Wang et al., 2022). Many methods such as chemical or fertilizer application, salt-resistant variety development, and plant genetic engineering have been employed for ameliorating such SS-induced impacts (Bui, 2013; Fatma et al., 2014). In recent studies, investigators have paid increasing attention to applying beneficial microbes to mitigate SS (Vaishnav et al., 2019).

Bacterial endophytes are widely distributed in plants, where they colonize intracellular and intercellular spaces. Both fungal and bacterial endophytes typically colonize and live within an individual host plant throughout its life cycle without causing any significant pathological symptoms (Ryan et al., 2008; White et al., 2019). The inoculation of endophytic bacteria into plants promotes the synthesis of compounds such as antioxidants, amino acids (AAs), and sugars and enhances photosynthesis in host plants alleviating SS (Diskit et al., 2018). Phytohormones produced by bacterial endophytes, including jasmonic acid (JA), abscisic acid (ABA), and indole acetic acid (IAA), promote development in their hosts (Singh et al., 2021; Roy Choudhury et al., 2022) while ACC-deaminase reduces ethylene production under SS (Ali et al., 2014). These microorganisms can also reduce plant SS by producing ROS-scavenging enzymes and increasing K^+^ absorption for counteracting Na^+^ while modulating relevant pathways (Fan et al., 2020). Other studies have reported the role of endophytic bacteria in mitigating plant SS by restoring plant biochemical and physiological states (Kumar et al., 2020). Endophytic bacteria also have important roles in modulating microbial community structures within plant rhizospheres (Cordovez et al., 2019). Due to the growth-promoting effect of endophytic bacteria on plants, more carbon is fixed and transferred to the rhizosphere, where it is released in the form of root exudates (Compant et al., 2010; Li et al., 2021). As a result, more beneficial microorganisms move toward the exudate, colonizing and multiplying within the rhizosphere and root nodules (Lugtenberg and Kamilova, 2009). Galaviz et al. (2018) inoculated mesquite (*Prosopis articulata*) with *B. pumilus* ES4, which improved the relative abundance of the beneficial bacteria *Paenibacillaceae* and *Bacillaceae* in the rhizosphere, thus contributing directly or indirectly to plant growth promotion. In addition, inoculation with the endophytic *Bacillus velezensis* JC-K3 in wheat (*Triticum aestivum* L.) changed bacterial and fungal diversities within the roots and reduced plant salt damage (Ji et al., 2021). *Enterobacter ludwigii* can survive at 15–42°C and pH 5–10 (Hoffmann et al., 2005). Khan et al. (2020) found that *E. ludwigii* generated IAA, gibberellins (GA) and organic acids, thus promoting plant development while enhancing its resistance. Nowadays, little is known about how *Enterobacter ludwigii* alleviates abiotic stress in plants, especially the mechanisms by which changes in microbial communities in the rhizosphere and root of plants regulate plant resistance to abiotic stresses are unclear.

Bermudagrass (*Cynodon dactylon*), a typical warm-season turf grass, is widely used in temperate and tropical regions of the world (Wei et al., 2022a). Turfgrass managers are using reclaimed water as an irrigation resource because of the decreasing availability and increasing cost of fresh water (Xiang et al., 2018). However, reclaimed water contains large amounts of salt ions, and its prolonged use can elevate the salt content of turfs, thereby inhibiting the growth of turfgrass (Liu et al., 2019). Nonetheless, the effect of bacterial endophytes on improving SS resistance of bermudagrass remains unclear. Our previous work isolated endophytic bacterium strain *E. ludwigii* B30 from *Paspalum vaginatum* roots in the salinization experimental site of the Teaching and Research Base in South China Agricultural University, Guangzhou, China (lat.23°30′15″N, long.113°80′21″E). and found that the inoculation of *E. ludwigii* B30 is an effective way to improve seed germination, root length, and seedling length of bermudagrass under salt stress (Zhao et al., 2021). The present work examined the role of *E. ludwigii*. B30 in mitigating high SS-induced unfavorable impacts on Bermudagrass plant development. Additionally, we carried out 16S rDNA amplicon sequencing to determine how *E. ludwigii* B30 affected the bacterial community structure in bermudagrass rhizosphere soils and roots. Understanding the effects of endophytic bacteria on the structures of root and rhizosphere bacterial communities associated with bermudagrass contributes to understanding the complicated interactive network between microbes and their host.

## Materials and methods

### Plant materials as well as growth conditions

Bermudagrass (*Cynodon dactylon*) seeds were obtained from the International Grass Industry Co. Ltd. in Tianjin, China. Seeds were immersed in 75% ethanol for 5 min to surface-sterilize them, washed 4 times in sterile distilled water, and planted into plastic pots (diameter 10 cm, height 10 cm; 20 g m^–2^ sowing rate) containing substrate (peat: sand 3:1, V/V). The plastic pots were sterilized at 121°C for 1 h in an autoclave on three successive days prior to setting up the experiment. Potted seedlings were cultured in a growth chamber [controlled at 30/27°C; 60% relative humidity; a 14 h/10 h light/dark cycle; 650 mmol m^–2^ s^–1^ photosynthetic active radiation (PAR)]. Pots were initially watered in the morning and evening to keep the soil moist. After seedling emergence, plants were watered one time a day until water drained from the bottoms of the pots to maintain soil water content and fertilized once weekly with half-strength Hoagland’s solution.

### Salt-tolerant endophytic bacteria

The salt-tolerant bacteria *Enterobacter ludwigii* B30 was isolated from *Paspalum vaginatum*. A salt tolerance test showed that B30 could grow normally on 500 mM NaCl medium. Before inoculation, 2 ml *E. ludwigii* B30 was activated in beef extract peptone liquid medium and shaking at 30°C for 10 h until OD_600_ reached 0.8.

### Bacterial inoculant for bermudagrass growth under salt stress

A randomized complete block experimental design was used, and there were two factors, each with two levels: (1) non-inoculation (E−) and inoculation with *Enterobacter ludwigii* B30 (E +), and (2) NaCl concentration of 0 and 250 mM. Each treatment was replicated three times.

E + treatment was inoculated in 28-days-old bermudagrass seedling roots with 20 mL of *E. ludwigii* B30 bacterial suspension was adjusted with 10 mM MgSO_4_ to a concentration of 10^9^ CFU mL^–1^. E− treatment was 20 mL of 10 mM MgSO_4_ solution. Three days after inoculation, all plants were trimmed to a height of 4 cm. To avoid salt shock, NaCl was added to the pots in a stepwise manner by increasing NaCl concentration to 50, 100, 150, 200 and 250 mM daily. Once the 250 mM NaCl content was achieved, it was maintained for 14 days. The day of bacterial inoculation of plants was designated day 0. At 21-days post-stress, root/shoot lengths and fresh/dry plant weights were measured, and plant samples were collected for analysis.

### Quantification of photosynthesis-related parameters

Chlorophyll fluorescence parameters were determined in the bermudagrass leaves following *E. ludwigii* B30 inoculation and salt treatments. We used a chlorophyll fluorometer imaging-PAM (Walz, Effeltrich, Germany) to determine chlorophyll fluorescence in the middle part of the first leaf from every seedling, collected at ambient temperature. After a 20 min adaptation in the dark, chlorophyll fluorescence was measured in the plant samples. We set the intensities of saturating and actinic lights at 7200 and 185 μmol m^–2^ s^–1^ PAR, respectively. In line with Murchie and Lawson (2013), quantum yields of regulated and non-regulated energy dissipation of PSII [Y(NPQ), Y(NO) separately] were measured, along with the maximum quantum yield of PSII (Fv/Fm), and the photochemical energy conversion efficiency via PSII Y(II). We calculated parameters qP and qN in equations 1 and 2 below (Rascher et al., 2000). The parameters of chlorophyll fluorescence were determined for 3 leaf sample sets (4 leaf samples from every set) from each pot on the day before harvest.


(1) $$qP=\left(F^{\prime }m-Fs\right)/\left(F^{\prime }m-F^{\prime }0\right)$$

(2) $$qN=\left(Fm-F^{\prime }m\right)/F^{\prime }m$$

where Fm and F’0 represent maximum and minimum fluorescence intensities of leaf samples adapted to darkness for a 30-min period; F’m is the maximum fluorescence of the light-adapted leaf sample, and Fs is real-time fluorescence of leaves.

### Determination of turf quality, relative water content, antioxidant enzyme activities and photosynthetic pigment content

For evaluating the health as well as the overall performance of bermudagrass, we measured turf quality (TQ) according to color, grass canopy uniformity, and density with a 1–9 point scale, with 1 indicating totally brown and dried plants, 9 indicating a green, dense, turgid canopy, and 6 indicating the minimum acceptable level (Supplementary Table 1) (Beard, 1973).

For measuring relative water content (RWC) in leaves, leaf samples were harvested and weighed to determine the fresh weight (FW). The leaf samples were then immersed in deionized water for a 24 h period at 4°C before removing the leaves from the water and determining the turgid weight (TW). Afterward, the leaves were dried for 72 h at 80°C in an oven before recording dry weight (DW). Leaf RWC was calculated using equation 4 (Barrs and Weatherley, 1962):


(3) (FW-DW)/(TW-DW)×100

Free proline levels were measured using the method of Bates et al. (1973). We homogenized the freshly collected leaves (500 mg) in sulfosalicylic acid (10 ml, 3% w/v) and filtered it through Whatman No. 2 filter paper. The filtered extract was mixed with glacial acetic acid and acid ninhydrin (2 mL each) and boiled at 100°C for 1 h. After retrieving 2 mL of toluene from the mixture, it was stirred for 15–20 s. Absorbance (OD) was later measured in the toluene blank at 520 nm, followed by isolation of the upper layer from the aqueous phase.

Superoxide dismutase (SOD) activity was measured with a xanthine oxidase T-SOD activity assay kit (Nanjing Jiancheng Bioengineering Institute, Nanjing, China). This determines the inhibitory effect of xanthine in producing superoxide anion free radicals under the catalysis of xanthine oxidase, thereby determining SOD activity. The produced superoxide radicals oxidized hydroxylamine for the generation of nitrite, which served as a developer for creating the purple color. OD values were determined in every reaction mixture at 550 nm. The amount of SOD needed to produce a 50% inhibition of nitrite reduction within the 1 mL reaction system was determined as one SOD activity unit (Beyer and Fridovich, 1987). We utilized an assay kit for detecting CAT activity (Nanjing Jiancheng Bioengineering Institute, Nanjing, China). CAT activity was measured as the reduction in OD value at 240 nm resulting from H_2_O_2_ decomposition. The enzyme volume inducing 1 mmol H_2_O_2_ decomposition/second at 25°C in a 1 g tissue protein sample was deemed to be one CAT activity unit. CAT and SOD activities (U/g protein, with U indicating enzymatic activity unit) were determined following manufacturer’s protocols (Rady et al., 2019).

Chlorophyll was extracted from freshly collected leaf samples (1 g) through suspension in 80% acetone (5 mL v/v). A spectrophotometer (UV-2800, Unico, Shanghai, China) was utilized to record extract OD values at 470, 646 and 663 nm. Carotenoid and chlorophyll levels were measured using equations in Lichtenthaler and Wellburn (1983).

### Determination of ion concentration and membrane stability

K^+^ and Na^+^ ions content in leaves and roots were measured using the method of Chang et al. (2018). Fresh leaves and roots were dried on filter paper, followed by an additional 2-days drying to constant weight at 70°C. K^+^ and Na^+^ ions in root and leaf dry matter (0.1 g) were obtained for subsequent analysis. Samples were mixed with an acid mixture (5 mL) containing HClO_4_ and HNO_3_ at a 1:4 ratio, followed by overnight incubation at 60°C and later introduction of 1 mL H_2_O_2_ and 4 mL HNO_3_ for 20 min at 220°C. After 4 h of cooling under ambient temperature, deionized water was added to dilute the extract, followed by injection into an Atomic Absorbance Spectrophotometer [AA-6650, Shimadzu (China) Co., Ltd., JP]. This work obtained ions from 3 plants from every treatment. No plant sample was used in the blank group.

To measure MDA content, 0.2 g of freshly collected leaves were homogenized in 5% trichloroacetic acid (TCA, 1.5 mL), and centrifuged at 14,000 g for 25 min. 0.5 mL aliquots of the supernatant were mixed with 20% TCA (1 mL) containing 0.5% thiobarbituric acid (TBA), heated for 30 min at 100°C, rapidly cooled and then centrifuged at 10,000 g for 10 min. The supernatant was used to measure OD values at 532 and 600 nm. MDA content was measured at the 155 mM^–1^cm^–1^ extinction coefficient after deducting non-specific absorbance (600 nm) (Heath and Packer, 1968).

Membrane permeability (MP) was measured based on the leaf electrolyte leakage (EL, determined as a percentage). An increase in EL indicates an increased MP value (Wei et al., 2022b). Freshly collected leaf blade samples (300 mg) from several pots were placed in a test tube containing distilled water (30 ml). After 12-h of shaking, electrical conductivity (E1) was measured using a conductivity meter (DDS-307; Zhengyi Technology Co., Guangzhou, China). Samples were then placed into boiling water for 1 h, and electrical conductivity (E2) was again measured at ambient temperature. EL was calculated using the formula:


(4) EL=(E1-E0)/(E2-E0)×100

where E0 indicates the electrical conductivity in distilled water (Lutts et al., 1996).

### Endogenous abscisic acid, indole acetic acid, and 1-aminocyclopropane-1-carboxylate measurement

Abscisic acid (ABA) and IAA content was measured by Phytodetek-ABA and Phytodetek-IAA Immunoassay kits (Agdia, Elkhart, IN) (Cluis et al., 2004; Vanderauwera et al., 2007). We determined phytohormone levels following manufacturer’s instructions. Shoots were immersed in liquid nitrogen and ground into powder. The powder (0.5 g) was suspended in the extraction solution [8 ml, containing 80% methanol, 0.5 g L^–1^ citric acid monohydrate together with 100 mg L^–1^ butylated hydroxytoluene (BHT)], followed by overnight stirring in the dark at 4°C followed by 20 min centrifugation at 1000 g and 4°C. The resulting supernatant was collected into another tube, vacuum dried, and dissolved in tris-buffered saline (TBS, 900 μL, pH 7.8) and 100% methanol (100 μL). ABA and IAA contents were then measured from the filtrate using the Phytodetek ABA/IAA enzyme immunoassay kit (Agdia Inc., Elkhart, IN).

To measure ACC concentrations in seedling tissues roots (1 g) were immersed in liquid nitrogen and ground to powder. ACC extraction was then carried out using 2 mg BHT-containing 80% methanol (5 ml) as the antioxidant, followed by 45 min incubation at ambient temperature. After 15 min centrifugation at 2000 g and 20°C, pellets were resuspended in 80% methanol (4 ml), followed by centrifugation. The resulting supernatant was evaporated to dryness with a rotary evaporator. ACC content was measured using the method of Madhaiyan et al. (2007). The residue was resuspended in distilled water (2 ml), and dichloromethane was added. In the upper aqueous phase, 0.5 mL was extracted and mixed with 80 mM HgCl_2_ (0.1 ml) in a test tube, followed by sealing using rubber septa. Thereafter, each tube had 0.2 ml of sodium hypochlorite solution added, followed by 8 min of incubation in a shaker.

### DNA extraction and high-throughput sequencing

Rhizosphere soil and root samples were collected at the end of salt stress treatments. Plants were collected and gently shaken to remove the loosely adhered soil after which rhizosphere soil samples were collected by removing the remnant soil with a fine sterile brush. The root samples were gently rinsed several times with tap water then washed with sterile water, followed by drying on sterilized filter paper. Rhizosphere soil and root samples were stored at −80 °C for DNA extraction. We isolated total DNA from around 0.5 and 0.1 g rhizosphere soils and root separately with the Soil DNA Kit (OMEGA, Shanghai) and plant DNA kit (Tiangen, Beijing) in line with manufacturer’s protocols. A Qubit Fluorometer was used to measure DNA quality using the Qubit dsDNA BR Assay kit (Invitrogen, Waltham, MA, USA). 0.1% agarose gel was utilized to test quality. All extracts were stored under 20°C. To conduct PCR amplification, the sequences of primers in the bacterial v3-v4 region (5′-CCGTCAATTCMTTTRAGTTT-3′) and (5′-GTGCCAGCMGCCGCGGTAA-3′) were applied. After amplification, products were subject to recovery and purification, fluorescence intensity was quantified, and a sequencing library was constructed. Finally, we used the Illumina HiSeq platform to conduct sequencing at Guangdong Magigene Biotechnology Co., Ltd. Guangzhou, China.

### Bioinformatics analysis

QIIME software (quantitative insights into microbial ecology, v1.8.0^1^) (Caporaso et al., 2010) was used to obtain high-quality clean tags. Typically, this retained sequences whose lengths were ≥ 160 bp, and fuzzy bases were eliminated. Sequences that had > 8 continuous identical bases and those with > 1 5′-primer base mismatches were also ruled out. Usearch (v5.2.236^2^) was utilized for checking and removing chimeric sequences after which the desired sequences were obtained. OTU clustering was carried out at a similarity level of 97%. Afterward, Silva databases (Quast et al., 2013) for bacteria were used to identify OTU classifications following clustering analysis.

### Statistical analysis

Data were collated with Microsoft Excel 2019. SPSS22.0 (SPSS Inc., Chicago, IL, United States) was used for the statistical analyses of treatment effects on the bermudagrass plant physiology. We applied one-way and two-way ANOVAs and Fisher’s LSD test to test significant differences (*P* < 0.05) between treatments in plant physiology indicators. Origin 8.0 software was employed to plot relative abundance maps showing bacteria whose mean relative abundances were >1%. LEfSe analysis was then conducted to analyze relative general abundances using the Galaxy online analysis platform.^3^ The R Project (v4.1.3) ggplot2 package and Bray-Curtis distance metric were used to perform principal coordinate analysis (PCoA), and β-diversity was visualized with the phyloseq package (Oksanen et al., 2007). Redundancy analysis (RDA) was conducted, and RDA diagrams were drawn using the R (v4.1.3) ggplot2 and vegan packages.

## Results

### Effects of *Enterobacter ludwigii* B30 on the growth of bermudagrass under salt stress

**FIGURE 1:**
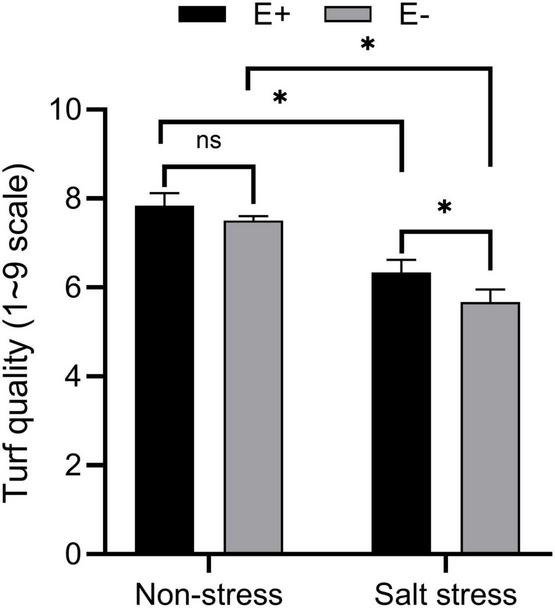
Effects of bermudagrass turf quality of inoculated and uninoculated plants under salt stress. Bars denote means ± SE of four treatment. “Non-stress” represents bermudagrass grown without NaCl condition; “Salt stress” represents bermudagrass under 250 mM salt conditions. “E +” represents the infection of *Enterobacter ludwigii*, “E−” represents the absence of *E. ludwigii.* “*” indicate significant differences at *P* < 0.05 and “ns” represents no significant difference.

Under SS, *E. ludwigii* B30 (E +) treated plants showed markedly different shoot lengths compared with non-treated control plants. Non-endophyte treated (E−) plants had a 13.40 cm shoot length, while E + plants had a 16.01 cm shoot length (Table 1). E + plants also had markedly increased root length (*P* < 0.05) compared with E− treated plants under SS. The fresh weight (FW) of shoot and root biomass increased in plants growing with E +. Under SS, E− plants had fresh weights of shoots and roots of 4.75 g and 1.14 g, whereas those of E + treated plants were 8.78 g and 1.68 g. Similarly, shoot and root dry weights (DW) were improved in plants in the E + treatment. SS exposure significantly decreased (*P* < 0.05) turf quality (TQ) of E + treated and E− treated bermudagrass. However, the TQ of the E + treated plants was greater than (*P* < 0.05) that of the E− treated plants in SS conditions (Figure 1 and Supplementary Figure 1).

**TABLE 1:** Influence of *Enterobacter ludwigii* B30 on shoot length, root length, shoot biomass and root biomass of bermudagrass in the presence and absence of salt stress.

Treatment	Shoot height (cm)	Root length (cm)	Shoot biomass (g FW)	Root biomass (g FW)	Shoot biomass (g DW)	Root biomass (g DW)
**Non-stress**						
E−	18.33 ± 0.21b	10.68 ± 0.36b	12.31 ± 0.35b	1.69 ± 0.15b	1.43 ± 0.03a	0.21 ± 0.01b
E +	20.11 ± 0.85a	13.33 ± 0.22a	15.94 ± 0.43a	2.06 ± 0.09a	1.66 ± 0.08b	0.26 ± 0.03a
**Salt stress**						
E−	13.40 ± 0.51d	6.17 ± 0.36d	4.75 ± 0.25d	1.14 ± 0.06c	1.11 ± 0.02c	0.03 ± 0.01d
E +	16.01 ± 0.19c	7.26 ± 0.19c	8.78 ± 0.21c	1.68 ± 0.11b	1.41 ± 0.08a	0.15 ± 0.02c
**ANOVA**						
E	***	**	***	***	***	***
Salt	***	***	***	***	**	***
E × Salt	***	**	**	***	ns	**

Difference in various growth parameters and comparative analysis. Average ± standard error from three separate replicates. “Non-stress” represents bermudagrass grown without NaCl condition; “Salt stress” represents bermudagrass under 250 mM salt conditions. “E +” represents the infection of E. ludwigii, “E−” represents the absence of E. ludwigii. Different letters means significantly different based on P < 0.05. “**,” “***” indicate significant differences at P < 0.01, and P < 0.001, respectively, and “ns” represents no significant difference.

Salt and *E. ludwigii* B30 did not significantly affect shoot dry weight (*P* > 0.05), but affected other plant growth indicators (Table 1).

### Physiological response following inoculation of *Enterobacter ludwigii* B30 in salt stress treated plants

**FIGURE 2:**
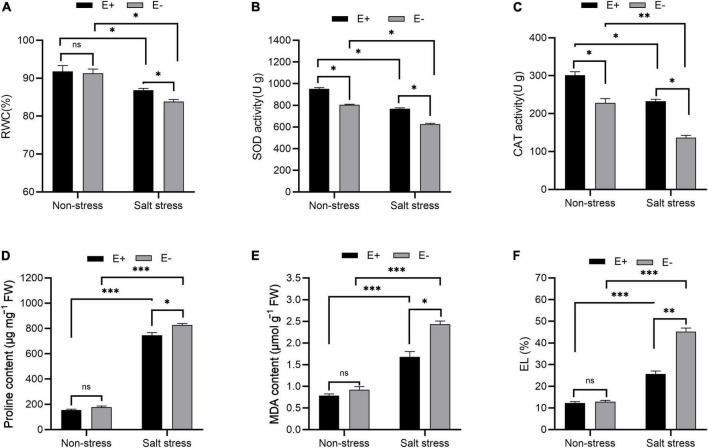
Physiological response. Relative water content (RWC) **(A)**, superoxide dismutase (SOD) activity **(B)**, catalase (CAT) activity **(C)**, proline content **(D)**, malonaldehyde (MDA) content **(E)** and electrolyte leakage (EL) **(F)** of bermudagrass with inoculated and uninoculated plants under salt stress. Bars denote means ± SE of four treatment. “Non-stress” represents bermudagrass grown without NaCl condition; “Salt stress” represents bermudagrass under 250 mM salt conditions. “E +” represents the infection of *Enterobacter ludwigii i*, “E−” represents the absence of *E. ludwigii.* “*,” “^**^”, “^***^” indicate significant differences at *P* < 0.05, *P* < 0.01, and *P* < 0.001, respectively, and “ns” represents no significant difference.

Salt stress (SS)-treated plants exposed to E + had increased RWC and decreased proline levels compared with controls. For E-SS treatment plants, the RWC was approximately 83%, and for E + plants, it was approximately 87% (Figure 2A). In E-SS treatment plants, proline levels were approximately 827 μg mg^–1^ FW, while in the E + treatment they were 745 μg mg^–1^ FW (Figure 2D).

Leaf SOD activity under SS significantly increased in E + plants compared with E−plants (726 U g^–1^ and 625 U g^–1^, respectively; *P* < 0.05; Figure 2B). The leaf CAT activity within plants under SS with E + treatment increased compared with the E− treatment (232 U g^–1^ and 136 U g^–1^, respectively; *P* < 0.05; Figure 2C).

MDA content and electrolyte leakage under SS decreased in the E + treatment in comparison with plants in the E− treatment (2.43 μmol g^–1^ FW and 1.68 μmol g^–1^ FW, respectively; Figure 2E). For plants under SS, an electrolyte leakage rate of 45% was seen from plants in the E− treatment, compared with 25% in plants in the E + treatment (Figure 2F).

### *Enterobacter ludwigii* B30 enhanced photosynthetic efficiency and protected photosynthetic pigments of bermudagrass under salt stress

**FIGURE 3:**
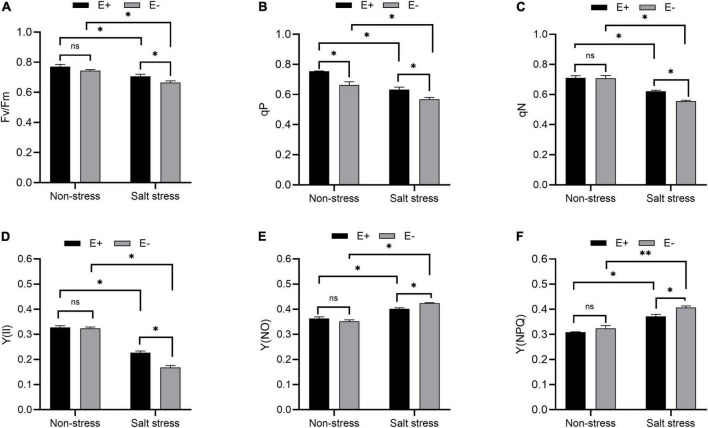
Chlorophyll fluorescence parameter. Optimal/maximal quantum yield of PS II [**(A)**; Fv/Fm], photochemical quenching [**(B)**; qP], non-photochemical quenching [**(C)**; qN], quantum yield of PSII [Y(II); **(D)**], Y(NO) **(E)** and Y(NPQ) **(F)** of bermudagrass with inoculated and uninoculated plants under salt stress. Bars denote means ± SE of four treatment. “Non-stress” represents bermudagrass grown without NaCl condition; “Salt stress” represents bermudagrass under 250 mM salt conditions. “E +” represents the infection of *Enterobacter ludwigii*, “E−” represents the absence of *E. ludwigii.* “*,” “^**^,” indicate significant differences at *P* < 0.05, and *P* < 0.01, and respectively, and ‘ns’ represents no significant difference.

The Fv/Fm ratio (maximum quantum yield of PSII), photochemical quenching (qP), and non-photochemical quenching (qN) in bermudagrass plants grown under salt stress with E + increased significantly compared with E− plants (Figures 3A–C; *P* < 0.05). PSII [Y(II)] quantum yield increased dramatically, whereas Y(NPQ) and Y(NO) decreased significantly in salt-stressed bermudagrass plants grown with E + compared with E− plants (Figures 3D–F; *P* < 0.05).

**FIGURE 4:**
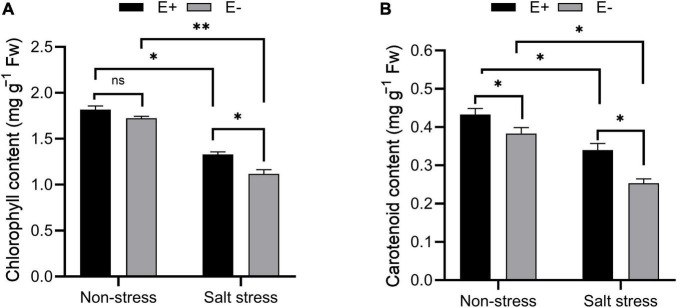
Estimation of photosynthetic pigments. Chlorophyll **(A)** and carotenoid **(B)** contents of bermudagrass with inoculated and uninoculated plants under salt stress. Bars denote means ± SE of four treatment. “Non-stress” represents bermudagrass grown without NaCl condition; “Salt stress” represents bermudagrass under 250 mM salt conditions. “E +” represents the infection of *Enterobacter ludwigii*, “E−” represents the absence of *E. ludwigii.* “*,” “^**^,” indicate significant differences at *P* < 0.05, and *P* < 0.01, respectively, and ‘ns’ represents no significant difference.

Chlorophyll and carotenoid content increased under salt stress in E + plants compared with E− plants (Figures 4A,B; *P* < 0.05). The chlorophyll and carotenoid contents were 1.17 and 0.23 mg g^–1^ FW in E− plants compared with 1.36 and 0.32 mg g^–1^ FW in E + plants.

### Inoculation of *Enterobacter ludwigii* B30 affected levels of abscisic acid, indole acetic acid, and 1-aminocyclopropane-1-carboxylate under salt stress

**FIGURE 5:**
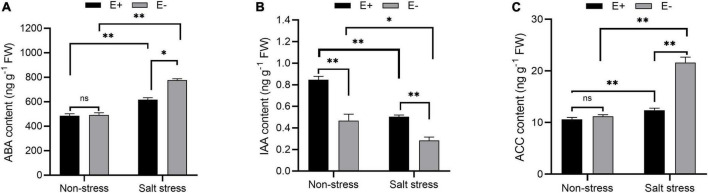
Changes in endogenous hormone content. Abscisic acid (ABA) **(A)**, indole acetic acid (IAA) **(B)** and 1-aminocyclopropane-1-carboxylate (ACC) **(C)** contents of bermudagrass with inoculated and uninoculated plants under salt stress. Bars denote means ± SE of four treatment. “Non-stress” represents bermudagrass grown without NaCl condition; “Salt stress” represents bermudagrass under 250 mM salt conditions. “E +” represents the infection of *Enterobacter ludwigii*, “E−” represents the absence of *E. ludwigii.* “*,” “^**^,” indicate significant differences at *P* < 0.05, and *P* < 0.01, respectively, and ‘ns’ represents no significant difference.

Without SS, ABA levels did not significantly change in bermudagrass leaves in E + compared with E− treatments (Figure 5A; *P* > 0.05). Under SS, the E + treatment decreased leaf ABA content by 21.3%, to 617.93 ng g^–1^. IAA levels in plants exposed to E + under SS were significantly higher than in E− plants (0.51 nmol g^–1^ FW and 0.27 nmol g^–1^ FW, respectively; *P* < 0.05, Figure 5B). ACC content in the leaves of bermudagrass under SS increased by 91.4% in E− plants compared with E + plants (*P* < 0.05), which led to increased ethylene production and inhibition of plant growth. Moreover, the ACC content of E + plants under SS decreased significantly compared with E− plants (12.39 ng g^–1^ FW and 21.48 ng g^–1^ FW, respectively; *P* < 0.05, Figure 5C).

### Ion concentration in response to bacterial inoculation under salt stress

In the absence of SS, the E + treatment did not significantly affect Na^+^ levels within bermudagrass leaf samples, but it significantly increased K^+^ content (*P* < 0.05). The E + treatment significantly reduced Na^+^ content in bermudagrass roots but significantly increased the K^+^ content (*P* < 0.05). Under SS, the E + treatment reduced the Na^+^ content in bermudagrass leaf and root samples while greatly increasing K^+^ content (*P* < 0.05). Under salt stress, the E + treatment decreased the Na^+^:K^+^ ratio within root and leaf samples compared with E− plants (Table 2).

**TABLE 2:** Influence of *Enterobacter ludwigii* B30 on Na^+^, K^+^, Na^+^: K^+^ ratio in roots and leaves, Na^+^ (root) to Na^+^ (leaf) ratio and K^+^ (root) to K^+^ (leaf) ratio of bermudagrass in the presence and absence of salt stress.

Treatment	Non-stress		Salt stress		ANOVA		
	E +	E−	E +	E−	E	Salt	E × Salt
**Root**							
Na^+^ (mg g^–1^)	1.34 ± 0.04d	2.62 ± 0.06c	24.49 ± 1.12b	32.10 ± 1.59a	***	**	**
K^+^ (mg g^–1^)	9.66 ± 1.21b	5.91 ± 1.02d	11.70 ± 0.87a	7.12 ± 0.43c	***	***	***
Na^+^/K^+^	0.14 ± 0.02d	0.44 ± 0.04c	2.09 ± 0.18b	4.50 ± 0.22a	***	***	***
**Leaf**							
Na^+^ (mg g^–1^)	1.63 ± 0.08c	1.62 ± 0.07c	14.47 ± 0.89b	19.97 ± 1.68a	***	***	***
K^+^ (mg g^–1^)	7.89 ± 1.02c	7.40 ± 1.31c	12.66 ± 1.78a	9.91 ± 1.32b	***	***	**
Na^+^/K^+^	0.21 ± 0.04c	0.22 ± 0.03c	1.14 ± 0.12b	2.02 ± 0.16a	***	***	***
**Root/leaf**							
Na^+^/Na^ +^	0.82 ± 0.09c	1.62 ± 0.23b	1.70 ± 0.18a	1.61 ± 0.21b	**	**	*
K^+^/K^ +^	1.22 ± 0.21a	0.80 ± 0.11c	0.93 ± 0.18b	0.72 ± 0.22d	**	**	*

Difference in ion uptake analysis. Average ± standard error from three separate replicates. “Non-stress” represents bermudagrass grown without NaCl condition; “Salt stress” represents bermudagrass under 250 mM salt conditions. “E +” represents the infection of E. ludwigii, “E-” represents the absence of E. ludwigii. Different letters mean significantly different based on P < 0.05. “*,” “**,” “***” indicate significant differences at P < 0.05, P < 0.01, and P < 0.001, respectively, and “ns” represents no significant difference.

Changes to the ratio of concentrations of Na^+^ (root) and Na^+^ (leaf) indicate that Na^+^ is translocated from leaves to roots in plants grown with E + under salt stress. However, this ratio was not significantly different among plants grown with E−. Under non-salt stress conditions, the ratio in plants grown with E + was significantly lower than those with E–. Under SS, the K^+^ (root) to K^+^ (leaf) ratio is indicative of K^+^ translocation from roots to leaves in plants grown with E +. The ratio in plants grown with E + was significantly higher than in those with E–.

### Bacterial community composition within rhizosphere and root

**FIGURE 6:**
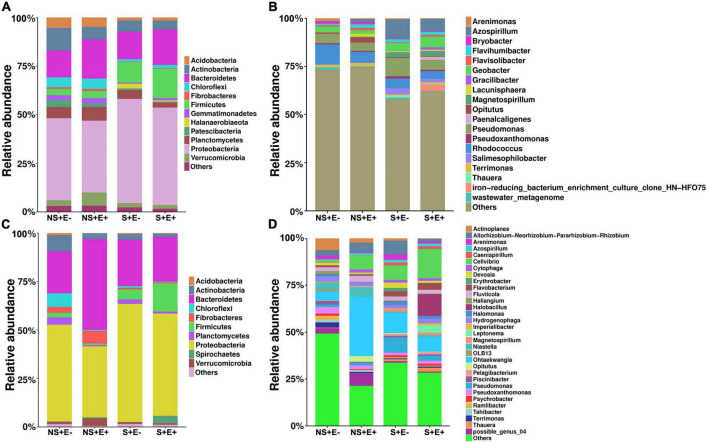
The relative abundance (at the phylum and genus level) of rhizosphere soil **(A,B)** and root **(C,D)** bacteria community under *E. ludwigii* B30 infection treatment and salt stress. (*n* = 3; “NS +” represents bermudagrass grown without NaCl condition; “S +” represents bermudagrass under 250 mM salt conditions. “E +” represents the infection of *E. ludwigii* B30, “E–” represents the absence of *E. ludwigii* B30).

Results of the analysis of rhizosphere soil bacterial phyla showed, under salt stress, that compared with the E− treatment, the E + treatment markedly increased Bacteroidetes (*F* = 4.193, *P* = 0.003), Firmicutes (*F* = 5.23, *P* = 0.023) and Chloroflexi (*F* = 3.803, *P* = 0.018) relative abundances (Figure 6); whereas it significantly decreased the relative abundances of Actinobacteria (*F* = 1.282, *P* = 0.005) and Proteobacteria (*F* = 4.186, *P* = 0.041). The relative abundance of Bacteroidetes (Figure 6A) in the NS + E + treatment increased significantly (*P* < 0.05) compared with the NS + E− treatment, reaching 20.1%. However, the relative abundance of Proteobacteria in the S + E + treatment increased significantly (*P* < 0.05) compared with the NS + E + treatment, reaching 53.6%.

In the analysis of the root bacterial phyla, under salt stress, the *E. ludwigii* B30 treatment (E +) markedly increased Firmicutes (*F* = 15.078, *P* = 0.038) and Spirochetes (*F* = 5.124, *P* = 0.029) relative abundances compared with the SS + E− treatment (Figure 6C) whereas the relative abundances of Actinobacteria (*F* = 2.333, *P* = 0.041) and Planctomycetes (*F* = 14.958, *P* = 0.018) significantly decreased. The relative abundances of Bacteroidetes, Fibrobacteres and Verrucomicrobia in NS + E + increased significantly (*P* < 0.05) compared with the NS + E− treatment. However, the relative abundance of Spirochetes and Firmicutes (Figure 6D) in the S + E + treatment significantly (*P* < 0.05) increased compared with the NS + E + treatment, reaching 3.9% and 14.6%, respectively; whereas the relative abundance of Actinobacteria in the S + E + treatment decreased significantly (*P* < 0.05) compared with the NS + E + treatment, reaching 1.6%.

Bacterial genera relative abundance in rhizosphere soil under SS in plants treated with *E. ludwigii* B30 (E +), compared with plants without *E. ludwigii* B30 (E−), showed increases for *Flavisolibacter* (*F* = 4.314, *P* = 0.023), *Geobacter* (*F* = 10.868, *P* = 0.004), *Gracilibacter* (*F* = 8.353, *P* = 0.005) and *Paenalcaligenes* (*F* = 13.939, *P* = 0.001), and decreases for *Arenimonas* (*F* = 11.011, *P* = 0.002), *Azospirillum* (*F* = 1.163, *P* = 0.014), *Pseudomonas*, *Pseudoxanthomonas* (*F* = 0.036, *P* = 0.003), *Salimesophilobacter* (*F* = 2.609, *P* = 0.013) and *Thauera* (*F* = 0.551, *P* = 0.001) (Figure 6B). The relative abundance of *Flavisolibacter*, *Lacunisphaera*, and *Opitutus* in the NS + E + treatment increased significantly (*P* < 0.05) compared with the NS + E− treatment, reaching 20.1%. However, the relative abundances of *Geobacter*, *Gracilibacter* and *Magnetospirillum* in S + E + treatment markedly (*P* < 0.05) increased compared with the NS + E + treatment.

Bacterial genera relative abundance in roots under SS in *E. ludwigii* B30 (E +) treatment compared with the S + E− treatment increased for *Actinoplanes* (*F* = 1.079, *P* = 0.001), *Cellvibrio* (*F* = 0.531, *P* = 0.021), *Halobacillus* (*F* = 15.972, *P* = 0.012), *Imperialibacter* (*F* = 5.031, *P* = 0.025), and *Pelagibacterium* (*F* = 8.071, *P* = 0.006), and decreased for *Allorhizobium-Neorhizobium-Pararhizobium-Rhizobium* (*F* = 4.281, *P* = 0.004), *Arenimonas* (*F* = 1.029, *P* = 0.016), *Erythrobacter* (*F* = 14.119, *P* = 0.022) and *Ohtaekwangia* (*F* = 12.082, *P* = 0.024) (Figure 3D). *Fluviicola* and *Ohtaekwangia* (Figure 6D) relative abundance in the NS + E + treatment increased significantly (*P* < 0.05) compared with the NS + E− treatment. The relative abundance of *Cellvibrio*, *Flavobacterium*, *Halobacillus*, *Imperialibacter* and *Leptonema* in the S + E + treatment increased significantly (*P* < 0.05) compared with the NS + E + treatment, whereas the relative abundance of *Allorhizobium-Neorhizobium-Pararhizobium-Rhizobium* in S + E + treatment dramatically (*P* < 0.05) decreased relative to NS + S +, reaching 1.5%.

### Rhizosphere and root bacterial community richness, diversity and beta diversity analysis

**FIGURE 7:**
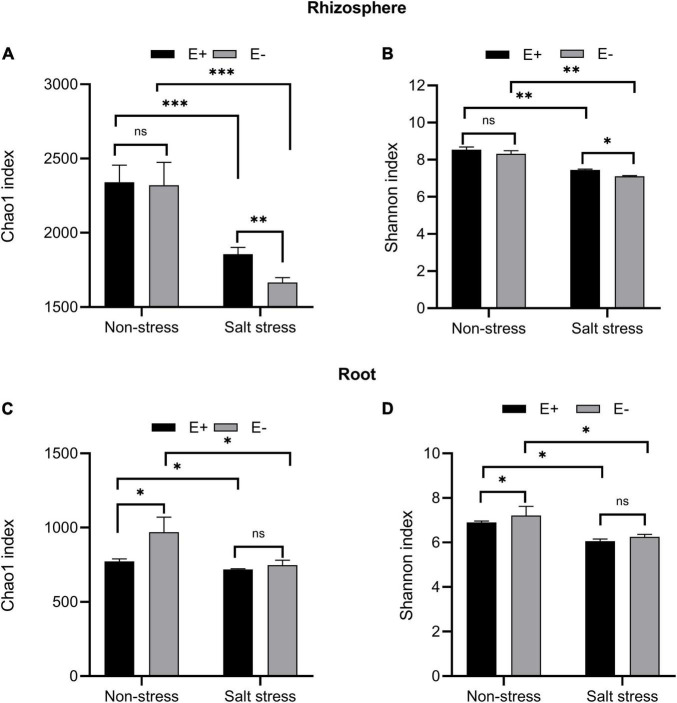
Alpha diversity. Bacterial diversity and richness in rhizosphere soil **(A, B)** and root **(C, D)**. Bars denote means ± SE of four treatment. “Non-stress” represents bermudagrass grown without NaCl condition; “Salt stress” represents bermudagrass under 250 mM salt conditions. “E +” represents the infection of *Enterobacter ludwigii*, “E–” represents the absence of *E. ludwigii.* “*,” “^**^,” “^***^” indicate significant differences at *P* < 0.05, *P* < 0.01, and *P* < 0.001, respectively, and ‘ns’ represents no significant difference.

Regardless of the endophyte treatment, salt stress greatly affected bacterial community richness measured by the Chao1 index in bermudagrass rhizosphere (*P* < 0.001, Figure 7A) and roots (*P* < 0.05, Figure 7C). Under Salt stress conditions, inoculation of *E. ludwigii* B30 significantly increased bermudagrass rhizosphere soil bacterial community richness compared with the E− treatment (*P* < 0.01), while the richness of the root bacteria community remained almost unchanged (*P* > 0.05).

Regardless of the endophyte treatment, SS dramatically affected bacterial community diversity, measured by the Shannon index, in bermudagrass rhizosphere soil (*P* < 0.01, Figure 7B) and roots (*P* < 0.05, Figure 7D). Under salt stress conditions, the E + treatment, compared with the E− treatment, significantly increased the bacterial community diversity in the bermudagrass rhizosphere soil (*P* < 0.05) but not in roots (*P* > 0.05).

For rhizosphere soil bacteria, PCoA plots showed different clusters for the E + and E–, salt stress and not salt stress treatments (Supplementary Figure 2A). PCoA1 and PCoA2 (first and second principal components) explained 29.86% and 13.65% of the variance, respectively. The root bacteria PCoA plots (Supplementary Figure 2B) showed separate clusters for E + and E− treatments (PCoA1 and PCoA2 explained 18.38% and 16.16% of the variance, respectively).

### LEfSe

**FIGURE 8:**
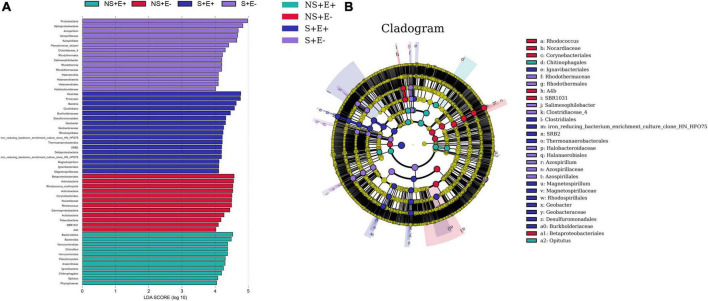
The taxonomic differences between treatments based on *E. ludwigii* B30 inoculation and salt stress in bermudagrass. The LEfSe map of bacteria including LDA **(A)** and Cladogram **(B)** in rhizosphere soil. LEfSe map is a linear discriminant analysis based on the composition of sample taxonomy according to different grouping conditions. In the four treatments, *P* < 0.05, LDA > 4 as the standard, communities with significant differences at each classification level are found to be represented by bar chart. Cladogram can intuitively show the difference information of samples at each classification level. “NS +” represents bermudagrass grown without NaCl condition; “S +” represents bermudagrass under 250 mM salt conditions. “E +” represents the infection of *Enterobacter ludwigii* B30, “E–” represents the absence of *E. ludwigii* B30.

We carried out LEfSe analysis to examine how salt stress and *E. ludwigii* B30 treatments affected bacterial taxa. There are Fifty-five bacterial taxa (from phyla to genera) with LDA scores > 4 (Figure 8A), twenty-nine of which were identified from order to genera among plants from the four treatments in bermudagrass rhizosphere soil (Figure 8B). Seven genus clades were found, including *Azospirillum*, *Pseudomonas_stutzeri*, and *Salimesophilobacter* from S + E–; *Geobacter* and *Magnetospirillum* from S + E +; Rhodococcus from NS + E–; and Opitutus from NS + E +.

**FIGURE 9:**
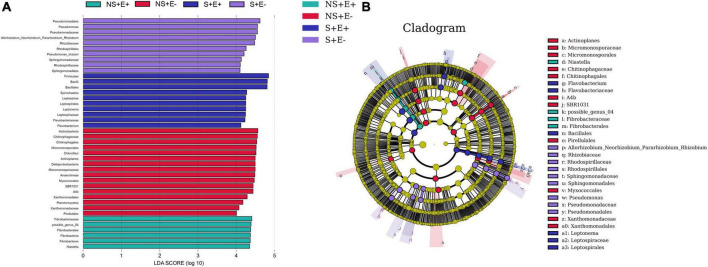
The taxonomic differences between treatments based on *E. ludwigii* B30 inoculation and salt stress in bermudagrass. The LEfSe map of bacteria including LDA **(A)** and Cladogram **(B)** in bermudagrass roots. LEfSe map is a linear discriminant analysis based on the composition of sample taxonomy according to different grouping conditions. In the four treatments, *P* < 0.05, LDA > 4 as the standard, communities with significant differences at each classification level are found to be represented by bar chart. Cladogram can intuitively show the difference information of samples at each classification level. “NS +” represents bermudagrass grown without NaCl condition; “S +” represents bermudagrass under 250 mM salt conditions. “E +” represents the infection of *Enterobacter ludwigii* B30, “E–” represents the absence of *E. ludwigii* B30.

There are Forty-two bacterial taxa (from phyla to genera) with LDA scores > 4 (Figure 9A), thirty of which were identified from order to genera among plants from the four treatments in bermudagrass roots (Figure 9B). Nine genus clades, including *Pseudomonas*, *Allorhizobium_Neorhizobium_Pararhizobium_Rhizobium*, *Pseudomonas_stutzeri*, and *Erythrobacter* from S + E–; *Spirochetes* and *Flavobacterium* from S + E +; *Chitinophagales* and *Actinoplanes* from NS + E–; and possible_genus_04 from NS + E +.

### Redundancy analysis

**FIGURE 10:**
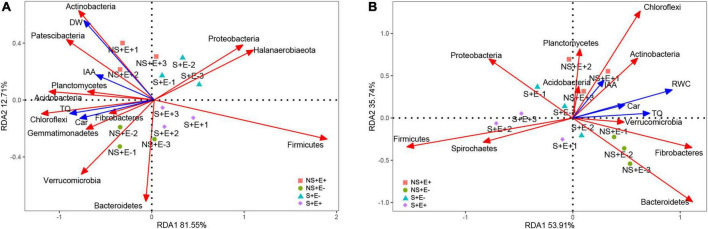
Redundancy analysis of relative abundance (phylum level) of rhizosphere soil bacteria and 4 treatments (NS + E–, NS + E +, S + E–, S + E +), and Environmental factors. Environmental factors include Total dry weigh (DW), Turf quality (TQ), indole acetic acid (IAA) and carotenoid (Car) [*n* = 3; **(A)**]. Redundancy analysis of relative abundance (phylum level) of root bacteria and 4 treatments (NS + E–, NS + E +, S + E–, S + E +), and Environmental factors. Environmental factors include Relative water content (RWC), Turf quality (TQ), indole acetic acid (IAA) and carotenoid (Car) [*n* = 3; **(B)**]. “NS +” represents bermudagrass grown without NaCl condition; “S +” represents bermudagrass under 250 mM salt conditions. “E +” represents the infection of *E. ludwigii* B30, “E–” represents the absence of *E. ludwigii* B30.

The first and second RDA axes among rhizosphere soil bacteria communities and plant indicators contributed 81.55% and 12.71% to the variance (Figure 10A). The plant DW (root + shoot) was positively correlated with phyla Actinobacteria and Patescibacteria. TQ was positively related to the phyla Chloroflexi, Fibrobacteres, and Planctomycetes. Car content showed positive associations with phyla Chloroflexi, Fibrobacteres, and Gemmatimonadetes. The first and second RDA axes in root bacteria communities and plant indicators explained 59.31% and 35.74% of the variance (Figure 10B), respectively. TQ was positively correlated with the phyla Verrucomicrobia, Fibrobacteres, and Bacteroidetes. The IAA content was positively correlated with the phyla Actinobacteria and Chloroflexi.

## Discussion

### Inoculation of *Enterobacter ludwigii* B30 enhances bermudagrass growth and affects abscisic acid, indole acetic acid, and 1-aminocyclopropane-1-carboxylate levels

Salinity directly affects soil biological and physiochemical characteristics and has adverse impacts on plant growth and yield. The salinity-induced harmful impact on plant development can be ascribed to specific ion toxicity, osmotic stress, nutritional disturbance, or combinations of these factors (Parida and Das, 2005; Ju et al., 2021). Studies have inoculated endophytic bacteria to alleviate SS within numerous plant species (Pal et al., 2021; Sofy et al., 2021). In this study, *E. ludwigii* B30 inoculation enhanced plant development in non-SS and SS conditions, which was manifested by higher shoot height, root length, shoot and root biomass (DW and FW) (Table 1) and TQ (Figure 1).

*Enterobacter ludwigii* has been shown to be an ACC deaminase-containing bacteria that can ameliorate drought tolerance in wheat (Gontia-Mishra et al., 2016). Studies have reported applying endophytic bacteria to ameliorate abiotic stresses such as NaCl stress through enhanced ACC deaminase activity (Lu et al., 2021; Sofy et al., 2021). We observed that the ACC content of *E. ludwigii* B30 inoculated bermudagrass was significantly lower than un-inoculated bermudagrass under SS (Figure 5). ACC is an immediate precursor of ethylene and under SS, plants characteristically generate increased amounts of ethylene (Barnawal et al., 2017). Ethylene restricts plant growth and proliferation until ethylene levels are reduced, and the SS condition is ameliorated. ABA participates in several physiological processes in plants, such as plant growth, stomatal aperture control, and stress responses to drought or salt stress (Fahad et al., 2015). This effect was evident in the present study as *E. ludwigii* B30 inoculations reduced ABA content within bermudagrass under SS. This may be associated with decreased plant ethylene content as it has been shown to affect plant ABA production (Rowe et al., 2016). Thus, ACC deaminase-containing bacterial inoculation could affect plant ABA levels. The application of *E. ludwigii* B30 reduced ABA levels within stressed plants, indicating that stress levels decreased within these plants. In the present study, ACC deaminase-containing bacteria reduced the adverse effects of harmful stresses by managing ethylene and ABA levels. Some researchers reported that endophytic bacteria inoculation increased ABA content in pea (*Pisum sativum*) under non-stress situations (Sofy et al., 2021). The rise in ABA content induces photosynthesis-influencing stomatal closure (Hamayun et al., 2010). We found that ABA levels did not significantly change in bermudagrass leaves in E + compared with E− treatments under non-stress conditions. We propose that the *E. ludwigii* B30 strains used by us may better balance phytohormones content and promote plant growth, which await further study.

*Enterobacter ludwigii* B30 inoculation promoted growth while enhancing resistance in NaCl stressed plants, in association with IAA generation and increased ACC deaminase activity. The longer root growth in plants inoculated with *E. ludwigii* B30 under salt stress and in the control plants was partially because of the isolate-generated IAA. The endophytic bacteria-generated phytohormones, in particular IAA are related to root initiation as well as enhanced adventitious and lateral root length, thereby helping nutrient absorption in host plants (Otlewska et al., 2020).

### *Enterobacter ludwigii* B30 modulates plant physiology in bermudagrass under salt stress while mitigating NaCl-mediated ionic disturbance

Decreased RWC is a characteristic osmotic stress response in plants. Our results show that after bacterial inoculation, RWC was elevated under SS. Ahmad et al. (2013) and Abd Allah et al. (2018) reported elevated RWC in chickpea (*Cicer arietinum* Linn.) and mung bean (*Vigna radiata*), respectively, following bacterial inoculation upon SS. Bacterial inoculation can alleviate the SS-induced inhibition of root growth while promoting effective root system development, thus facilitating increased water absorption from deeper in the soil in plants under stress (Marulanda et al., 2010). Abiotic stresses such as SS can induce ROS production within plants. ROS typically react with essential compounds in plants, such as cell membranes, proteins, and lipids, thereby causing oxidative stress (OS) (Wang and Huang, 2019). To combat ROS-induced OS, plants produce a range of antioxidative enzymes such as catalase (CAT) and superoxide dismutase. SOD has been recognized as the first line of defense to resist ROS by enhancing ROS scavenging through the catalysis of O_2_^–^ dismutation into O_2_ and H_2_O_2_ (Wei et al., 2022b). Likewise, CAT can eliminate excess plant superoxide radicals, thereby protecting against OS, promoting the accumulation of dry matter, and improving plant resistance (Ahmad et al., 2022). Our results show CAT and SOD activities markedly decreased within *E. ludwigii* B30 inoculated bermudagrass compared with non-inoculated bermudagrass under SS (Figure 3). These results agree with the study of Masmoudi et al. (2021), where endophytic halotolerant *Bacillus velezensis* FMH2 inoculation reduced the OS-mediated harmful impacts in tomato (*Lycopersicon esculentum*).

Oxidative stress (OS) causes lipid peroxidation damage to cell membranes, thereby altering permeability and increasing cellular electrolyte leakage and malondialdehyde content (Alexander et al., 2020). In this study, EL and MDA content was elevated in SS treatments, indicating damage to cell membranes in saline conditions. *E. ludwigii* B30 inoculation decreased MDA content and EL (Figure 3), suggesting that endophytic bacteria helped to resist the harmful NaCl-induced impact on the intact plant cell membrane. Baek et al. (2020) showed similarly that endophytic bacteria inoculation decreased the EL and MDA content in salt-stressed rice (*Oryza sativa* L.).

It is challenging to maintain cellular viscosity and turgidity in plants upon SS. To overcome this, plants produce osmolytes/osmoprotectants, which assist in their survival under harsh conditions while maintaining intracellular water retention (Zulfiqar et al., 2020). As an AA, proline can serve as an osmoprotectant that exerts a range of effects upon different abiotic stresses and as a scavenger to eliminate hydroxyl free radicals (Chen et al., 2020). Sofy et al. (2021) found that endophytic bacterium *B. subtilis* inoculation increased the proline content in salt-stressed pea, which may be caused by a change in proline-metabolizing enzymes’ activity. Additionally, it has been proposed that higher proline concentrations might facilitate the scavenging of ROS to protect proteins and other important biomolecular structures and prevent adverse effects of SS (Abd Allah et al., 2018). However, we noticed that under SS, plants with *E. ludwigii* B30 inoculation exhibited reduced proline contents. However, we observed that bacteria-inoculated plants had low proline content under SS. Decreased in proline content in bacteria-inoculated and salt-stressed plants, indicate that the synthesis of osmotically active substances was not enhanced despite the presence of the salt. This finding has been reported by many scientists and was connected with the alleviation of salt stress (Jha et al., 2011; Rojas-Tapias et al., 2012). We propose that the *E. ludwigii B30* strains we utilized activate a mechanism distinct from the promotion of proline synthesis that is in charge of reducing salt stress. The plant’s response to salt stress’ osmotic component is proline production. Since bacterial inoculation prevents the rise in proline content, it is possible that bacteria reduce osmotic stress (Szymańska et al., 2020). As a result, the osmotic adjustment is not necessary since the synthesis of osmoprotective chemicals, such as proline, is constrained, which lowers the energy cost of acclimating to salt.

As a photosynthesis parameter, Fv/Fm can be used for detecting plant stress (Maxwell and Johnson, 2000). Fv/Fm measures disturbances in the thylakoid structure at PSII donor sites or photosynthetic electron transport (Zhang et al., 2018). The qP metric, which represents the fraction of PSII open centers, can be used to evaluate energy dissipation by means of PSII electron transport (Rohacek, 2002). Fv/Fm and qP values were higher in *E. ludwigii* B30 inoculated bermudagrass than in non-inoculated bermudagrass under salt stress, indicating that *E. ludwigii* B30 was important for helping the plant maintain optimum photosynthesis. The qN processes associated with heat dissipation can protect photosynthesis when more light energy is absorbed than can be utilized (Müller et al., 2001). qN is measured by decreased PSII antenna efficiency resulting from the great luminal [H + ] and xanthophyll cycles, while the latter is related to the proton gradient closely associated with PSII–PSI electron transport (Kramer et al., 2004). In this study, carotenoid levels (Figure 4) and qN (Figure 3) in bermudagrass increased after inoculation with *E. ludwigii* B30 compared with non-inoculated bermudagrass under salt stress, suggesting that *E. ludwigii* B30 alleviated the SS-induced inhibition of the lutein cycle while significantly increasing photosynthetic protection.

Light energy absorbed by chlorophyll is processed in 3 pathways, namely, photosynthesis (photochemistry), dissipation as heat, and re-emission as light-chlorophyll fluorescence-sometimes referred to as qN (Oxborough and Baker, 1997; Macedo et al., 2008). Following Kramer et al. (2004),


Y(II)+Y(NPQ)+Y(NO)=1

Y(II) represents photochemical yield, and the PSII-mediated photochemical energy conversion efficiency, Y(NPQ) is the down-regulation mediated dissipation yield, and Y(NO) is the additional non-photochemical loss yield. Y(NO) measures the non-light-mediated (basal or dark) quenching process. Y(NPQ) is also a measure of chlorophyll, carotenoid, and photoinhibition, and it may just represent changes in those rapidly reversible processes. The above three parameters compete with each other so that an increase in one induces a reduction in the other two. Therefore, by determining chlorophyll fluorescence yield, data regarding changes in heat dissipation and photochemistry efficiency can be acquired. This is determined by the elevation of pH differences in and outside of the thylakoid membrane (ΔpH) occurring, in the case of light saturation, during linear photosynthetic electron transport. In addition, it is related to xanthophyll cycle activation (Crouchman et al., 2006). Our results show Y(II) in *E. ludwigii* B30 inoculated bermudagrass PSII, following SS treatment, decreased compared with non-inoculated bermudagrass; whereas Y(NO) and Y(NPQ) increased compared with non-inoculated bermudagrass (Figure 3), suggesting that the *E. ludwigii* B30 application significantly increased the PSII photochemical efficiency under salt stress. Based on alterations of pigment levels (Figure 4), we observed that *E. ludwigii* B30 positively affected chlorophyll, carotenoid level, and photosynthetic performance in bermudagrass under SS. Endophytic bacteria are reported to have a similar protective effect on photosynthetic pigments within sugar beet (*Beta vulgaris* L.) under SS (Shi et al., 2010). Such increases in chlorophyll and carotenoid content may suggest that *E. ludwigii* B30 application increased the absorption of light, protected bermudagrass plant photosynthetic systems, and enhanced the capacity of PSII for processing photochemistry under salt stress.

We found that the ratio of Na^+^(root) to Na^+^(leaf) markedly decreased in un-inoculated bermudagrass compared with *E. ludwigii* B30 inoculated bermudagrass under SS, indicating that *E. ludwigii* B30 inoculation restricted Na^+^ translocation from the roots to aerial parts of bermudagrass, thereby restricting toxic Na^+^ accumulation within photosynthetic tissues (Porcel et al., 2016). Sahu et al. (2021) showed similarly that endophytic bacteria inoculation restricted Na^+^ translocation from the roots to shoot in salt-stressed tomato. Under SS, excess Na^+^ ions will compete against K^+^ (the other macronutrient), inducing nutritional and metabolic disturbance and exerting adverse impacts on plants. An increased K^+^/Na^+^ ratio is an indicator of an efficient SS resistance mechanism (Etesami and Glick, 2020). Plants in the present study also exhibited a markedly decreased Na^+^ (root, leaf) concentration and increased K^+^ (root, leaf) concentration after *E. ludwigii* B30 application under SS, which suggest that endophytic bacteria help to enhance plant development through altering ion selectivity while maintaining an increased K^+^ content in comparison with Na^+^. These results agree with the study of Shi et al. (2022), where endophytic strain *B. megaterium* ZS-3 inoculation improved the K^+^/Na^+^ ratio in *Arabidopsis thaliana* to alleviate SS. We found increased Na^+^ and decreased K^+^ levels within uninoculated SS-exposed plants compared with controls, whereas there was no such effect in plants inoculated with *E. ludwigii* B30. The decline in Na^+^ levels following *E. ludwigii* B30 inoculation could result from bacterial exopolysaccharides binding to cations (in particular Na^+^) within roots, preventing cation transfer into leaves and alleviating SS within plants (Ashraf et al., 2004).

### *Enterobacter ludwigii* B30 impacted rhizosphere and root bacterial communities in bermudagrass

Rhizosphere effects (Smalla et al., 2001), the alteration to the composition and number of microbial communities, and their impacts on above-and below-ground plant development were seen within bermudagrass following *E. ludwigii* B30 inoculation. *E. ludwigii* B30 inoculation led to increases in bermudagrass rhizosphere soil bacterial community richness and diversity (Figure 7). The inoculation of *E. ludwigii* B30 had a clear impact on bermudagrass rhizosphere bacterial species. These results are similar to studies on *Enterobacter cloacae* HG-1 strains which were shown to affect the rhizobacterial community structure and improve the salt tolerance of wheat (Ji et al., 2020). Our results show that *E. ludwigii* B30 affected the structure of bacterial communities within rhizosphere soils and roots (Supplementary Figure 2); however, the rhizosphere soil bacterial community richness and diversity increased more than the root bacterial communities (Figure 7). Bacterial community diversity has also been shown to decline from rhizosphere soil to roots within wheat (Donn et al., 2014) and rice (Edwards et al., 2015). This may be linked to the effect of the soil nutrient state affecting bacterial communities in soils. Typically, the root system has an important effect on linking plants with soils. Secondary metabolites can be released into the soil by plant roots, which can provide energy for microbial communities in rhizosphere soils (Vacheron et al., 2013). Bacterial communities closely connected with roots represent the simplest form (Donn et al., 2014). Conversely, other research reports that the root bacterial community richness and diversity dramatically increased within soils in *Ammophila breviligulata*, thriving in sand dunes. It is possible that in sandy soils within dune ecosystems, root exudates supply essential resources to root bacteria (Bell-Dereske et al., 2017).

**FIGURE 11:**
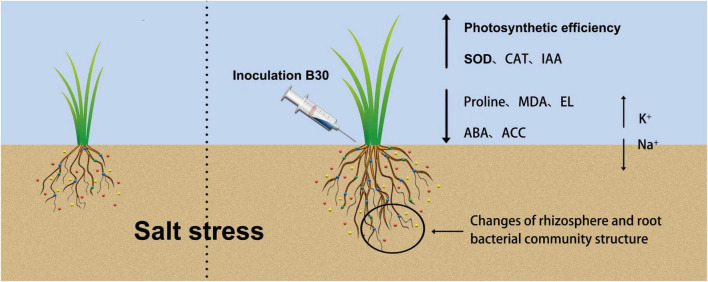
A graphical abstract describing the mechanism of endophytic bacterium strain B30 enhancing salt tolerance of bermudagrass we followed to accomplish this work. Under salt stress, B30-associated bermudagrass shows an increase in shoot biomass production as compared to non-B30 bermudagrass. Inoculation of B30 changed rhizosphere and root bacterial community structure, recruited some beneficial bacteria in rhizosphere and root, then enhanced Photosynthetic efficiency, SOD, CAT, and IAA content, also decreased proline, MDA, EL, ABA, and ACC content. Besides, B30 balanced Na^+^ and K^+^ concentration between shoot and root of bermudagrass to alleviate the damage caused by salt stress.

Endophytic bacteria can construct and maintain the key soil bacterial populations to promote plant growth; additionally, they may change the bacterial community in plant roots, which in turn affects plant growth (Ji et al., 2021). Here, we investigated the abundance of taxa in bermudagrass rhizosphere soils and roots and hypothesized that *E. ludwigii* B30 jointly promote the growth of bermudagrass under salt stress by regulating plant physiology and altering the rhizosphere and root bacterial communities (Figure 11). At the phylum level, Proteobacteria, Bacteroidetes, and Firmicutes were the predominant phyla detected within E+ and E− bermudagrass rhizosphere soils. Proteobacteria are known for recycling nutrients and improvement in soil fertility and plant growth (Beckers et al., 2017). Bacteroidetes play crucial roles in recycling organic matter (Fernández-Gómez et al., 2013). Firmicutes are associated with the decomposition of plant organic matter (Pasalari et al., 2020). Additionally, known copiotrophs, such as Bacteroidetes and Chloroflexi, are associated with nutrient concentration (Trivedi et al., 2013). The relative abundance of Bacteroidetes, Firmicutes, and Chloroflexi in *E. ludwigii* B30-inoculated bermudagrass rhizosphere soil significantly increased compared with non-inoculated bermudagrass under salt stress (Figure 6). Combined with the RDA result, TQ was positively related to Chloroflexi levels in rhizosphere soil (Figure 10A). TQ of bermudagrass in the E + treatment was significantly higher than in the E− treatment, suggesting the possible role of Chloroflexi in increasing rhizosphere soil nutrient content and plant organic matter decomposition. We also noted that Acidobacteria and Actinobacteria levels in rhizosphere soil were positively associated with TQ and DW, and they have been shown elsewhere to be associated with soils with nutrient deficiency (Beckers et al., 2017). The IAA content was positively related to the Actinobacteria in roots (Figure 10B). Some genera in the phylum Actinobacteria, such as *Streptomyces* and *Nocardia*, can be used as beneficial bacteria to promote plant growth. *Streptomyces* has been shown to exhibit favorable PGP characteristics, which solubilized phosphates and phytates, and produced siderophores and IAA (Alotaibi et al., 2022). In bermudagrass roots, inoculation of *E. ludwigii* B30 markedly elevated *Actinoplanes*, *Cellvibrio*, *Halobacillus*, *Imperialibacter*, and *Pelagibacterium* under salt stress (Figure 6). Of these bacterial genera, *Halobacillus and Pelagibacterium* are genera of bacteria with halophilic properties (Chen et al., 2009; Xu et al., 2011). *Imperialibacter* has the ability to hydrolyze casein and starch (Wang et al., 2013).

Thus, these *E. ludwigii* B30 and salt-induced rhizosphere soil and root beneficial bacteria may enhance bermudagrass growth and improve salt tolerance in some specific mechanisms, such as the improvement of the nitrogen cycle and nutrient uptake and phytohormones production. These await further study. The rhizosphere and root bacterial community might contribute to improving stress tolerance for plants (Dai et al., 2019). In further study, we will propagate and inoculate beneficial endophytic bacteria into plants and analyze the relationship between and soil physi-chemical properties and rhizosphere and root microbial community under salt stress. Managing rhizosphere and root microbes and maintaining the balance of beneficial and harmful microbes in soil and plant is an important part of improving plant tolerance to abiotic stresses.

## Conclusion

This work investigated how an *Enterobacter ludwigii* B30 strain obtained from *Paspalum vaginatum* affected bermudagrass growth under salt stress. *E. ludwigii* B30 improved the osmotic adjustment, biomass accumulation, and photosynthetic efficiency of bermudagrass, as well as selective ion absorption capacities. In addition, *E. ludwigii* B30 markedly altered bacterial community structure in the rhizosphere and roots of bermudagrass. Only limited conclusions can be drawn from the present study because only one endophyte strain and plant host were used; more investigations are warranted to analyze the rhizosphere-root community interactions together with salt tolerance in plants. Our findings help to explain how bermudagrass rhizosphere and root microbiota respond to endophytic bacteria inoculation. Our results suggest that *E. ludwigii* B30 promotes plant development and may serve as an inoculant for reducing SS damage in plants.

## Data availability statement

The datasets presented in this study can be found in online repositories. The names of the repository/repositories and accession number(s) can be found below: National Center for Biotechnology Information (NCBI) BioProject database under accession number PRJNA858230. Any queries should be directed to the corresponding author(s).

## Author contributions

JZ and TL conceived and initiated the project. HW and WH performed the experiments. ZL and HW performed the material preparation and data collection. LG conducted the bioinformatic analysis. HW wrote the first draft of the manuscript. All authors commented on previous versions of the manuscript, contributed to the study conception and design, and read and agreed to the published version of the manuscript.

## Conflict of interest

The authors declare that the research was conducted in the absence of any commercial or financial relationships that could be construed as a potential conflict of interest.

## Publisher’s note

All claims expressed in this article are solely those of the authors and do not necessarily represent those of their affiliated organizations, or those of the publisher, the editors and the reviewers. Any product that may be evaluated in this article, or claim that may be made by its manufacturer, is not guaranteed or endorsed by the publisher.

## Footnotes

1
http://qiime.org/

2
http://www.drive5.com/usearch/

3
http://huttenhower.sph.harvard.edu/galaxy/

## Supplementary material

SUPPLEMENTARY FIGURE 1 Morphological difference in different treatments. “NS +” represents bermudagrass grown without NaCl condition; “S +” represents bermudagrass under 250 mM salt conditions. “E +” represents the infection of *E. ludwigii* B30, “E−” represents the absence of *E. ludwigii* B30.

SUPPLEMENTARY FIGURE 2 Principal coordinates analysis (PCoA) of rhizosphere soil **(A)** and root **(B)** bacterial communities at operational taxonomic units (OTUs) level based on the Bray–Curtis dissimilarities under *E. ludwigii* infection treatment and salt stress. (*n* = 3; “NS +” represents bermudagrass grown without NaCl condition; “S +” represents bermudagrass under 250 mM salt conditions. “E +” represents the infection of *E. ludwigii*, “E–” represents the absence of *E. ludwigii* B30).
