# Supplementary figures and images for: Salt-tolerant endophytic bacterium Enterobacter ludwigii B30 enhance bermudagrass growth under salt stress by modulating plant physiology and changing rhizosphere and root bacterial community
Source: Front Plant Sci. 2022 Aug 2;13:959427. doi: 10.3389/fpls.2022.959427 (PMC9380843; doi:10.3389/fpls.2022.959427)

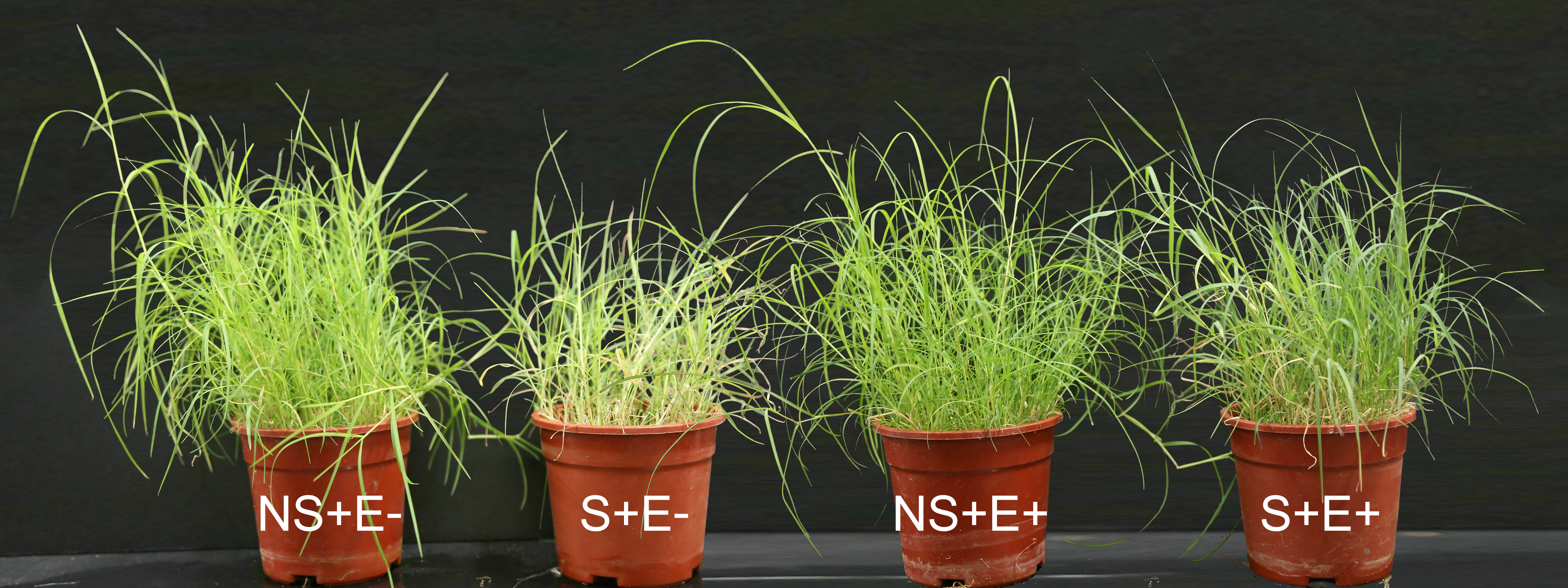

Supplement: SUPPLEMENTARY FIGURE 1 — Morphological difference in different treatments. “NS +” represents bermudagrass grown without NaCl condition; “S +” represents bermudagrass under 250 mM salt conditions. “E +” represents the infection of E. ludwigii B30, “E−” represents the absence of E. ludwigii B30. [file Image_1.JPEG]

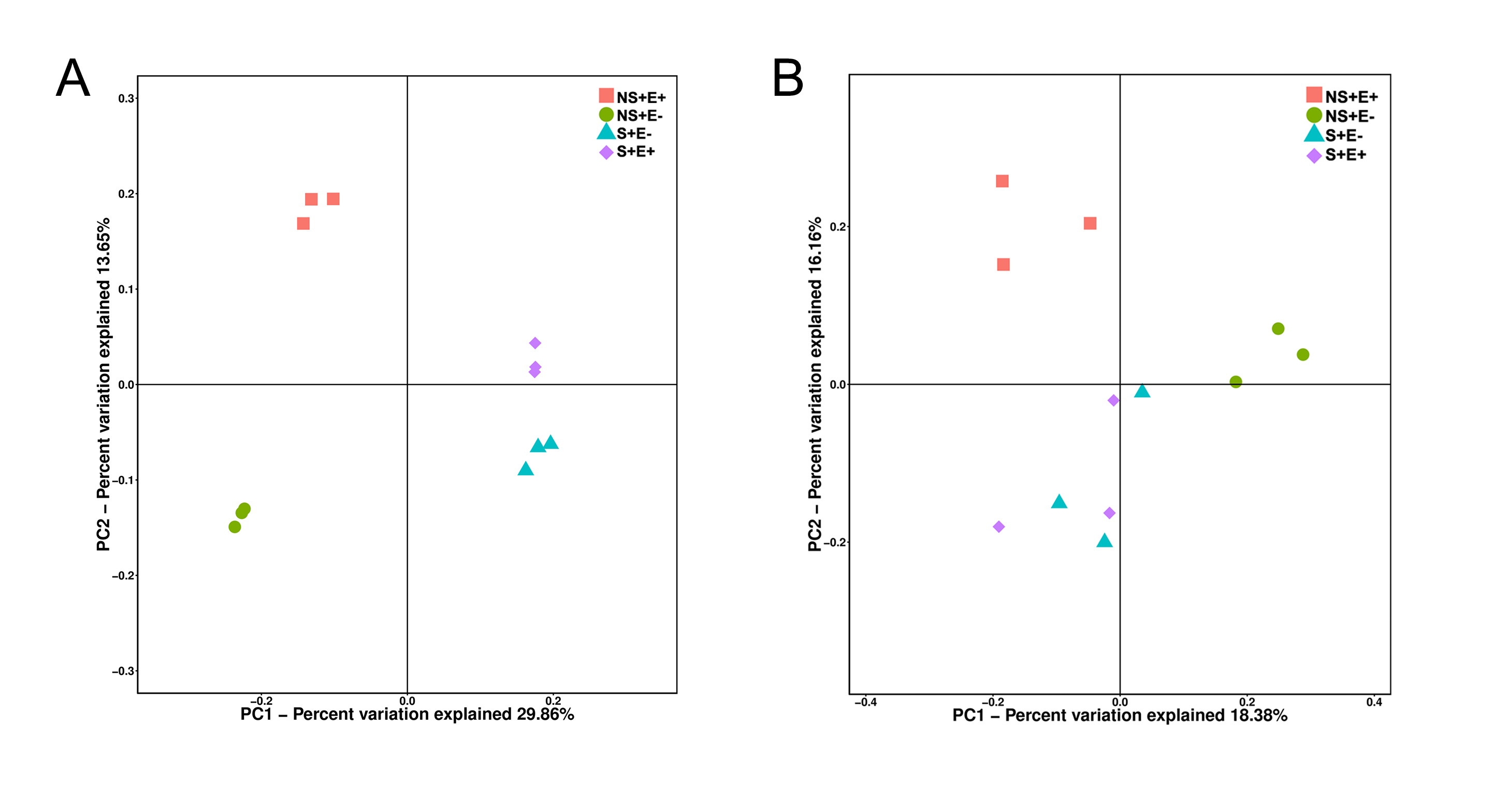

Supplement: SUPPLEMENTARY FIGURE 2 — Principal coordinates analysis (PCoA) of rhizosphere soil (A) and root (B) bacterial communities at operational taxonomic units (OTUs) level based on the Bray–Curtis dissimilarities under E. ludwigii infection treatment and salt stress. (n = 3; “NS +” represents bermudagrass grown without NaCl condition; “S +” represents bermudagrass under 250 mM salt conditions. “E +” represents the infection of E. ludwigii, “E–” represents the absence of E. ludwigii B30). [file Image_2.tif]
