# Supplementary material for: Salt-tolerant endophytic bacterium Enterobacter ludwigii B30 enhance bermudagrass growth under salt stress by modulating plant physiology and changing rhizosphere and root bacterial community
Source: Front Plant Sci. 2022 Aug 2;13:959427. doi: 10.3389/fpls.2022.959427 (PMC9380843; doi:10.3389/fpls.2022.959427)
Supplement: Supplementary file 3 [file Table_1.docx]

| Index | Grade range | Score |
| --- | --- | --- |
| Density | <50% | 1-3 |
|  | 50%～80% | 3-5 |
|  | 80%～100% | 5-6 |
|  | 100% coverage, sparser to very dense | 6-9 |
| Color | Dormant or yellowing | 1 |
|  | Much dead leaves, a few green | 1-3 |
|  | Much green, few dead leaves | 3-5 |
|  | Light green | 5-7 |
|  | darker green | 7-9 |
| Texture | Leaf width5-10 mm | 1-4 |
|  | Leaf width3-5 mm | 4-6 |
|  | Leaf width1-3 mm | 6-8 |
|  | Leaf width<1 mm | 8-9 |
| Uniformity | 50% balding | 1 |
|  | Very uniform | 9 |

Table S1 Evaluation level table of turf quality.

Weight: Color 20%; density 30%; texture 20%; uniformity 20%, the sum of the four scores is the turf quality (TQ) score.
